# Structural Formation of UHMWPE Film Tracked by Real-Time Retardation Measurements during Uniaxial/Biaxial Stretching

**DOI:** 10.3390/ma11112292

**Published:** 2018-11-15

**Authors:** Yoshinori Hashimoto, Shotaro Nishitsuji, Takashi Kurose, Hiroshi Ito

**Affiliations:** 1 Department of Organic Materials Science, Graduate School of Organic Materials Science, Yamagata University, 4-3-16 Jonan, Yonezawa, Yamagata 992-8510, Japan; nishitsuji@yz.yamagata-u.ac.jp (S.N.); takashi.kurose@yz.yamagata-u.ac.jp (T.K.); ihiroshi@yz.yamagata-u.ac.jp (H.I.); 2 Toyo Seiki Seisaku-syo, Ltd., 4-23, Ukima 5-chome, Kita-ku, Tokyo 115-0051, Japan

**Keywords:** uniaxial/biaxial stretching, retardation, birefringence, molecular orientation, stress-optical rule

## Abstract

This work reports on an experimental study of the stretching of ultra-high molecular weight polyethylene (UHMWPE) film in various uniaxial/biaxial stretching modes at various temperatures and stretching speeds. We examined the stress-birefringence relationship as a stress-optical rule (SOR) under uniaxial stretching and evaluated the stress-optical coefficient (SOC). Wide-angle X-ray diffraction (WAXD) measurements were applied to evaluate the contribution to birefringence of the crystalline and amorphous phases and to characterize stretching modes. In simultaneous biaxial stretching, the melting temperature (*T*_m_) proved critical to structural formation. We applied thermal analysis techniques and tensile testing to evaluate higher order structures after each stretching mode.

## 1. Introduction

Long-chain structures of semi-crystalline polymers have been used to create films with advanced properties. An understanding of the structural evolution of polymer film during stretching is essential to establishing ideal processing conditions, including temperature, stretching speed, draw ratio and stretching mode. Stretching of polymer film changes the crystallinity and the structure of crystalline polymers; these changes are more complex than for amorphous polymers.

Film stretching speed in industrial process nowadays reaches 400 m/min for polymers such as polypropylene (PP) and polyethylene terephthalate (PET) which can provide high production rate. However, it is an important task to make it compatible with the required properties of stretched film. Fast stretching speed generates sudden structural formation which narrows the suitable processing window. Insight into different types of stretching modes helps in controlling processing window and understanding the nature of the film. In laboratory research, on-line structure evaluations during film stretching have been studied to understand higher order structures in addition to off-line evaluation.

Recent studies indicate that UHMWPE film has been focused as an application in the field of lithium ion battery separator used in computer and communication applications [1,2]. However, while there are many reports on polymers such as PP and PET, still few reports exist on fast stretching of UHMWPE film. In UHMWPE film processing, solvent is usually added to resolve the difficulty of processing due to very high viscosity. Structural evaluations of biaxially stretched UHMWPE film without solvent have been studied by Uehara et al. [3]. Properties of UHMWPE and its relationship with stretched films in various conditions were also studied [4,5,6]. Stretching speeds conducted in reported studies were of the order of 0.1 m/min. Our motivation is to investigate structural formation of UHMWPE film under wider range of stretching speed and various stretching modes by on-line retardation measurement method.

Evaluations of birefringence, wide angle X-ray diffraction (WAXD), infrared spectroscopy and other measures are used to investigate higher order structures. Birefringence, which arises from the polarity of polymer molecules and molecular orientation, is an optical property defined by difference in the refractive index in orthogonal directions and indicates average orientation, including both the crystalline and amorphous phases. Investigations of the linear relationships at the onset of stretching between birefringence and stress, known as stress-optical rule (SOR), helps to illuminate the process of structural formation. Hassan et al. [7,8,9] discuss on-line measurements of birefringence and the development of higher order structures in investigations of mechano-optical behavior of PET films during stretching, reporting that SOR continues to apply up to the onset of strain hardening. Simultaneous measurements of stress and birefringence had been conducted by Kotaka et al. [10] in an elongational flow on low-density polyethylene blended with UHMWPE, confirming that stress-optical coefficient (SOC) was independent either of strain rate, stress, molecular weight and its distribution, and showed agreement with the value in reported studies [11,12]. Ryu et al. [13] have shown that SOR does not hold at low temperature close to glass transition temperature (*T*_g_) or at high strain rates due to the contributions of glassy stress. In our previous study [14], we investigated SOC of cyclic olefin copolymer films stretched at temperatures above *T*_g_, SOC values were found to approach the photo-elastic coefficient (PEC) under fast stretching speed.

In uniaxial stretching modes, the free-width and constrained-width stretching are known. Free-width stretching occurs as the width of the film shrinks and has been studied as a fundamental stretching mode. Relationship between draw-ratio and strengthening in mechanical properties was investigated by Gao et al. [15], the orientation behavior drawn to different draw-ratios was studied using deformation models by Bandeira et al. [16]. In contrast to free-width stretching, constrained-width stretching maintains constant width by clamping the sides of the film. This can be regarded as a type of biaxial stretching, since the polymer chains tend to orient toward the transverse direction (TD). Meng et al. [17] studied the effect of constraint in the TD, that it can provide broader stretching window as compared to free-width stretching. Ward et al. [18] investigated the deformation behavior in both free-width and constrained-width uniaxial drawings and showed it can be described by the deformation of a molecular network.

Biaxial stretching modes includes simultaneous and sequential biaxial stretching and are effective methods to obtain films with enhanced properties [19]. In general, simultaneous biaxial stretching makes it possible to achieve isotropic properties for the machine direction (MD) and for the TD. However, it requires complex stretching equipment and generally leads to limited production rates. In crystalline polymers, biaxial stretching is performed by breaking the crystalline structure and tie molecules. Thus, polymers of low crystallinity have a wide processing window. In sequential biaxial stretching, the initial stretching along the MD results in alignment parallel to the MD, resulting in strain-induced crystallization. With the second stretching along the TD, the elongation of the amorphous phase dominates compared to the crystalline phase. Types of orientation by different stretching modes were defined and classified by Heffelfinger et al. [20], which has been commonly referred in understanding of stretching modes.

Investigations of structure development under various stretching modes have been studied on various polymers, revealing the attainable properties of films. Ajji et al. [21] investigated the biaxial stretchability, developed structure, molecular orientation and shrinkage of linear low-density polyethylene (LDPE). Cakmak et al. [22] investigated the effects of stretching mode on the crystalline texture of polylactic acid (PLA) films. Under simultaneous biaxial stretching, PLA films showed in-plane isotropy with poor crystalline order. Under sequential biaxial stretching, development of oriented crystallization was confirmed at first stretching in the MD. Second stretching in the TD showed destruction of the former crystalline structure in the MD, establishing a second growth of oriented but poorly ordered crystalline structure. Kojima et al. [23] investigated biaxial oriented films of PE produced by blow extrusion. In biaxial stretching of film, there have been significant patent [19], concerning biaxial oriented polyethylene film with improved optical and sealability properties. 

In the current study, we used the newly developed stretcher combined with a retardation-measuring high-speed camera able to track retardation as on-line measurements under fast stretching speed up to 60 m/min, a speed comparable to industrial process. The effects of temperature, stretching speed, and draw-ratio (DR) on the structural development of UHMWPE film under different stretching modes were studied. Retardation and birefringence of the stretched films were measured using polarizing microscope as off-line measurements. WAXD measurements were carried out to evaluate crystallinity and orientation, the contribution of stretching mode to birefringence is discussed. Thermal analyses and tensile tests were performed to assess the structure of the stretched UHMWPE films.

## 2. Materials and Methods

### 2.1. Materials

We examined a UHMWPE film (Saxin NEWLIGHT^®^ #13W [24]) in this study. This film has *M*_w_ of 5.5 million g/mol. Raw UHMWPE particles are compressed and skived to a thickness of 130 µm. The material has a white semi-transparent appearance. The surface is very smooth. The density of the material is 0.94 g/cm^3^; the melting temperature (*T*_m_) is 137 °C. The degree of polymerization is about 10 times that of high-density polyethylene (HDPE). The film features extremely long linear chain structure and maintains its form beyond *T*_m_, permitting stretching across a wider stretching temperature window. Differential Scanning Calorimetry (DSC) scans identified a crystallinity of 59%.

### 2.2. Stretching Process

**Figure 1:**
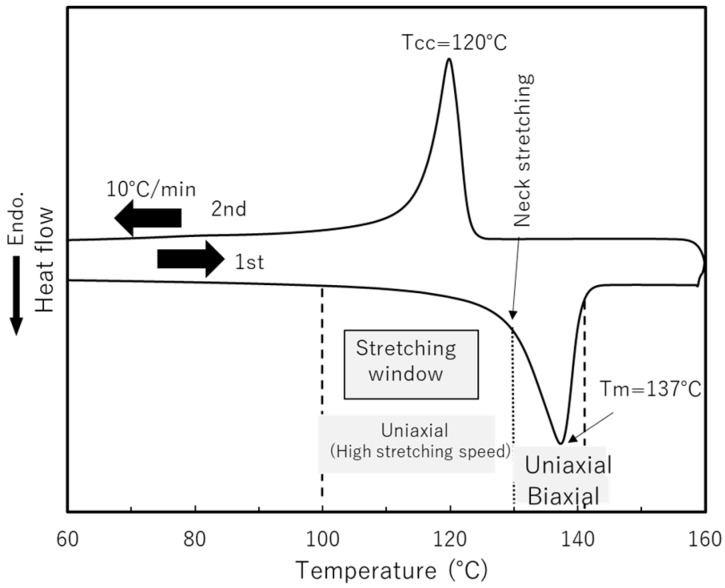
DSC measurement results for sample film showing stretching window in the experiment.

**Figure 2:**
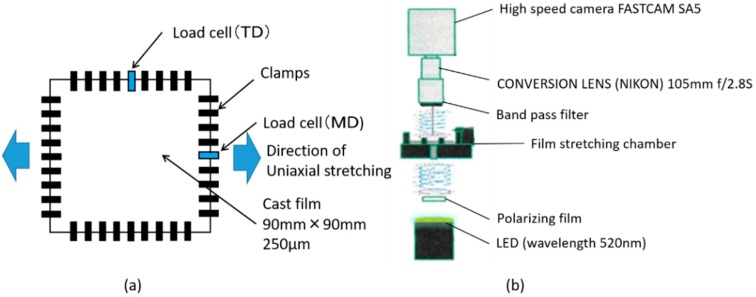
Schematic diagram of (**a**) film stretcher and (**b**) high-speed camera used for real time retardation measurements.

Figure 1 and Table 1 show DSC scans of the sample film and stretching window from this study. Figure 2 shows the film stretcher used, combined with high-speed camera. The sample film was moved by a pneumatic device to the clamping position in the chamber. The chamber was controlled to settle to equilibrium temperature, with a heater blower located underneath the clamping position. The preheating time before stretching was 3 min. Stretching temperatures ranged within the 100–140 °C range. Stretching modes included uniaxial free/constrained width stretching and simultaneous/sequential biaxial stretching. True stress was calculated by dividing engineering stress by instantaneous sectional area. Note that certain additional stretching conditions were tested to confirm the validity of our discussion.

### 2.3. Retardation and Birefringence Measurements

We arranged the film stretcher by attaching a high-speed camera (Photron Limited, Tokyo, Japan, FASTCAM SA5) enabling to track retardation of the film during stretching at high temporal resolution. The camera is provided with micro-scale polarizing elements and a pixel reading circuit. Monochromatic wave is emitted through polarizing film toward the film stretching chamber, where birefringence of the film produces polarized light. Result was obtained as convolution of retardation within the quarter of the wavelength range, therefore the degree of convolution was tracked and multiplied by 130 nm. While retardation shows distribution of a certain degree, we focused on the center of the film and an average value along about 1 cm width was evaluated.

For off-line measurements, polarizing microscope (Olympus Corporation, Tokyo, Japan, BX51-P) was used. Thick Berek compensator was installed to measure retardation of suitable range. The relationship between birefringence and retardation is:

Δ*n* = |*n_MD_* − *n_TD_*| = *R/d* (1)

where *d* is thickness and *n_MD_* and *n_TD_* are refractive indices along *MD* and *TD*, respectively. In crystalline polymer, Δ*n* was assumed to obey Stein’s addivity law [25]:

Δ*n* = *X_c_ f_c_* Δ*n_c_* + (1 − *X_c_*) *f_a_* Δ*n_a_* (2)

where first term on the right-hand side is contribution from crystalline phase, composed of crystallinity *X_c_*. degree of orientation *f_c_* and intrinsic birefringence of crystalline phase Δ*n_c_*. Second term is contribution from amorphous phase, where ratio of amorphous phase is derived from (1 − *X_c_*). Here, form birefringence was neglected and Δ*n_c_* = 0.058 was used [25]. These values were evaluated by WAXD analysis. Stress-optical constant (*SOC*) is given by:

*SOC* = Δ*n*/σ (3)

where σ is the true stress.

### 2.4. Wide-Angle X-ray Diffraction(WAXD)

Wide-angle X-ray diffraction (WAXD) measurements of the obtained stretched films were performed using X-ray diffraction instrument (Rigaku Corporation, Tokyo, Japan, Rigaku Micro). The Cu-Kα measurement (wavelength = 1.54 Å) radiation from an anode operating at 40 kV and 30 mA was used to detect the crystalline phase. Stretched films were cut and stacked to a thickness of about 0.3 mm in order to obtain sufficient accuracy. The orientation of crystalline phase was calculated via applying the azimuthal scan through the diffraction angle with respect to the selected plane and the ratio of half-width of diffraction peak was used for calculation. 1D-WAXD profiles were obtained from circularly integrated intensities of 2D-WAXD image acquired. Subsequently, decomposing the peaks of 1D-WAXD profiles into crystalline and amorphous phase as:

*X_c_* = ∑*A_c_*/(∑*A_c_* + ∑*A_a_*) (4)

where *A_c_* and *A_a_* are the fitted areas of crystalline and amorphous phase, respectively.

### 2.5. Light Transmittance

We measured light transmittance dispersions of the stretched films using customized light transmittance measuring equipment (Lambda Vision Inc., Kanagawa, Japan) within range of 350–1050 nm wavelength, which correspond to wavelength range of visible light. Software (Lambda Vision Inc., Kanagawa, Japan, ColorLabIV-LCD Ver.5.51) was used for analysis.

### 2.6. Thermal Analysis

#### 2.6.1. Differential Scanning Calorimetry (DSC)

We evaluated the crystallinity and the melting temperature *(T*_m_) of UHMWPE by DSC (TA Instruments, New Castle, DE, USA, Q200) at 30–160 °C at a heating rate of 10 °C/min.

#### 2.6.2. Dynamic Mechanical Analysis (DMA)

Dynamic mechanical analyzer (TA Instruments, New Castle, DE, USA, RSAIII) was used to measure the viscoelasticity of the stretched films. Heating rate was 2 °C/min in 40–160 °C range.

#### 2.6.3. Thermal Mechanical Analysis (TMA)

Thermal mechanical analyzer (TA Instruments, New Castle, DE, USA, Q400 TMA) was used. The film was cut into strip and clamped in the temperature controlling chamber. 0.02 N force was applied to the film during the measurement.

### 2.7. Mechanical Property Measurements

Dumbbell specimen was punched out from the stretched films by the dumbbell cutter DIN 53504-S3. Tensile testing machine (Toyo Seiki Seisaku-syo, Ltd., Tokyo, Japan, Strograph VG) was used to perform engineering stress-strain measurements with the strain rate of 50 mm/min.

## 3. Results and Discussion

### 3.1. Retardation and Birefringence Measurements

**Figure 3:**
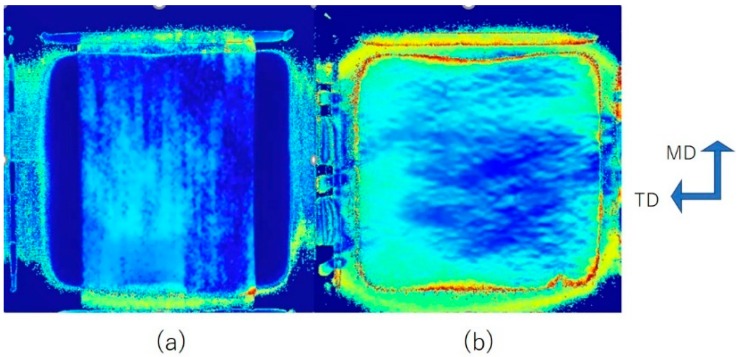
Retardation image of uniaxial: (**a**) free-width (**b**) constrained-width stretching along the MD at DR = 4 with stretching speed of 1 m/min.

Figure 3 shows retardation distribution image of uniaxial film stretching. Scale for the images is 9 cm by 9 cm. We can see that in free-width uniaxial stretching, retardation is fairly uniform. Whereas in constrained-width stretching, retardation reflects stress concentration at clamping point, and pleat like deformation along TD can be observed. The effect of constraint become apparent in the following results.

**Figure 4:**
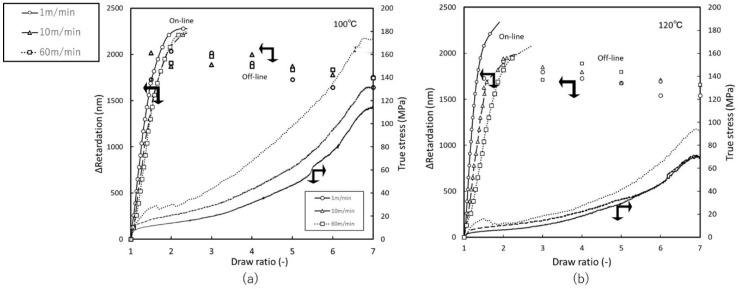
True stress and retardation vs. draw ratio of free-width uniaxial stretching at: (**a**) 100 °C (**b**) 120 °C.

Figure 4 shows the stress-strain curves and retardation behavior for uniaxial free-width stretching at 100 °C and 120 °C. The retardation results denote values relative to the preheated state, at which absolute retardation changes from null. It should also be noted that after DR = 2, decrease in retardation was difficult to track by on-line measurement, as retardation integration assumes monotonical behavior, thus we omitted beyond DR = 2 from the graphs. However, we observed relaxation after the end of stretching at each DR and confirmed that no significant relaxation was occurring. We can see that retardation reaches a maximum value by DR = 2, then decreases slowly. From the starting point of DR = 1, dependence on stretching speed becomes apparent at 120 °C. In the previous study of amorphous polymer [8], the relationship between stretching temperature and speed is such that lower temperatures and faster stretching produce comparable behavior. Here we can confirm same nature of dependence on temperature and stretching speed. However, variation range in SOC is narrower in stretching at 100 °C. This may be because polymer crystalline molecules at lower temperatures are more rigid, impeding structural change even at fast deformation rates. Stretching at 130 °C exhibited neck formation until DR = 4; the thickness of the stretched portion remained constant. Stress at 130 °C was too low, and the emergence of wrinkles on the surface of the films presented problems; thus, here we discuss only temperatures below this point.

**Figure 5:**
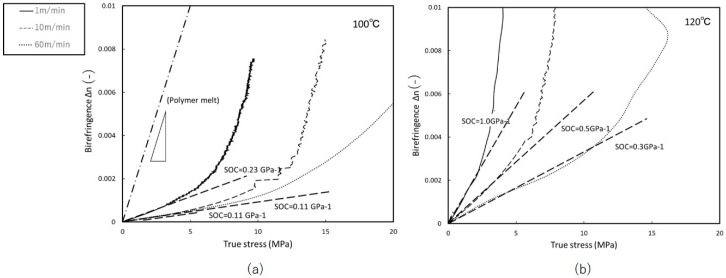
Birefringence vs. true stress of uniaxial free-width stretching at: (**a**) 100 °C (**b**) 120 °C.

Figure 5 shows the relationship between birefringence and true stress with free-width uniaxial stretching. We see that higher temperatures move the SOC closer to the value for the melt state. The SOC for high-density linear polyethylene melted at *T* = 423 K was reported to be 2.35 GPa^−1^ by Janeschitz [12], 1.3 GPa^−1^ was reported by Koyama et al. [11] for low-density polyethylene at molten state. Our results above give SOC = 0.11–1.0 GPa^−1^, which is comparable to accepted research.

**Figure 6:**
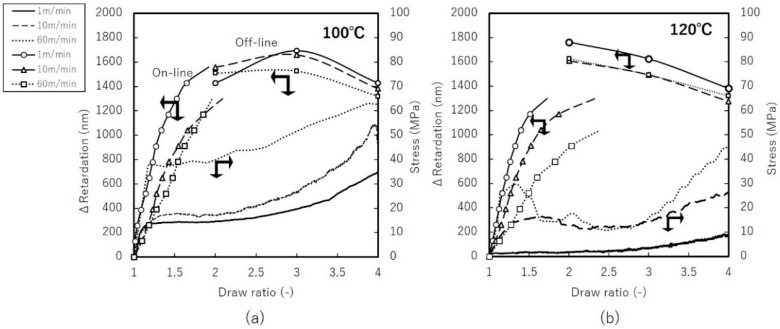
True stress and retardation vs. draw ratio of uniaxial constrained-width stretching at: (**a**) 100 °C (**b**) 120 °C.

**Figure 7:**
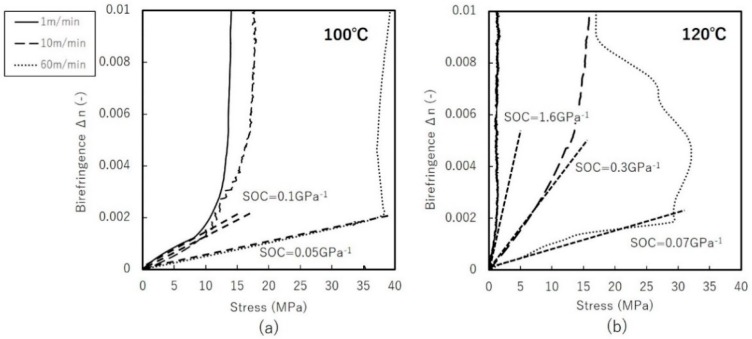
Birefringence vs. true stress of uniaxial constrained-width stretching at: (**a**) 100 °C (**b**) 120 °C.

Figure 6 and Figure 7 present the same argument for uniaxial constrained-width stretching. Constraints along TD resulted in lower stress compared to uniaxial free-width stretching. The dependence of birefringence on stretching speed becomes more apparent compared to Figure 4. In uniaxial constrained-width stretching, the SOC was found to be in the range of 0.05–1.6 GPa^−1^, a broader range than for uniaxial free-width stretching. This suggests that the stretching window for constrained-width stretching allows more control of the structural formation.

**Figure 8:**
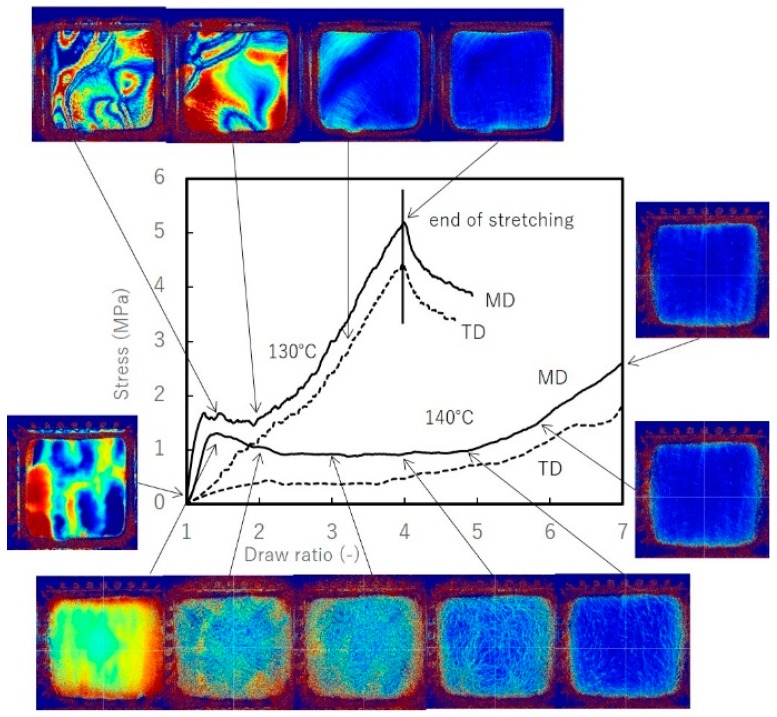
Stress vs. draw ratio of simultaneous biaxially stretched films at 130 °C and 140 °C with stretching speed of 1 m/min.

Figure 8 shows the relationship between the stress-draw ratio and retardation image at each DR. Scale for the images is 9 cm by 9 cm. At 130 °C, preheated film exhibits an unstable retardation distribution, which remains unchanged even after changing preheating times. As stretching begins, we observe deformation characterized by significant distortion until DR = 2.5 × 2.5. This behavior is similar to neck forming deformation in uniaxial stretching at 130 °C, in which stretched and non-stretched regions are intermingled in-plane. By DR = 3 × 3, stretching is completed and results in a uniform isotropic film with gloss and clarity. Compared to stretching at 140 °C, stress declines when temperatures are above *T*_m_. The boundaries of the raw UHMWPE powder expand as DR increases. In the early stages until DR = 2 × 2, we observe anisotropic retardation, followed by in-plane isotropic optical behavior.

**Figure 9:**
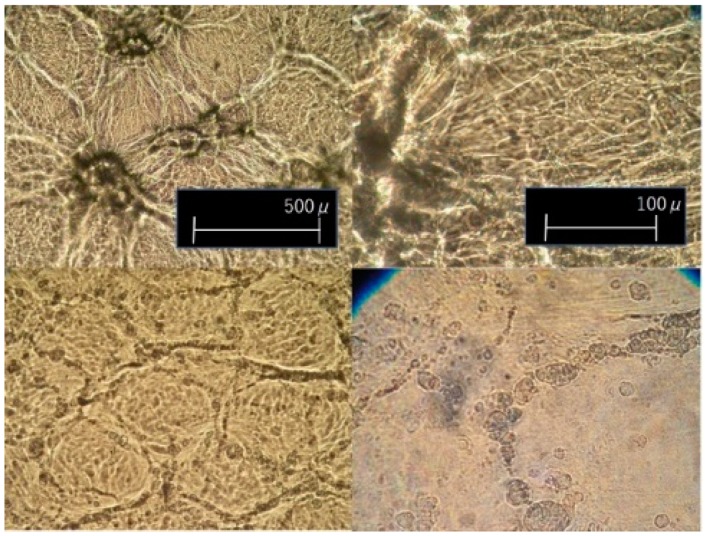
Optical micrographs of simultaneously biaxially stretched films at stretching speed of 1 m/min, DR = 4 × 4. (**upper**) 140 °C (**lower**) 130 °C.

Observations by optical micrograph provide more insight. Figure 9 shows optical micrographs of biaxially stretched films. In biaxially stretched films, traces of the boundaries of the raw particles are visible as well. Between the original boundaries, we see a fibrillar oriented form. However, as mentioned earlier, their optical properties and thus their morphology are quite different, depending on stretching temperature. As will be shown in WAXD analysis, stretching at 130 °C lowers crystallinity and results in higher transparency. At temperatures above 140 °C, fibrillar portion gains more mobility, resulting in a greater remaining non-stretched region as stress decreases. Tiny holes begin appearing in the fibrillar region at low DR; thus, stretching at higher temperature only degrades the quality of the film and narrows the stretching window. It is noted that in uniaxially stretched films, the boundaries of the raw UHMWPE powder are observed at intervals of about 100–130 µm, elongated along the stretching axis and exhibiting affine deformation.

### 3.2. Wide-Angle X-ray Diffraction (WAXD)

**Figure 10:**
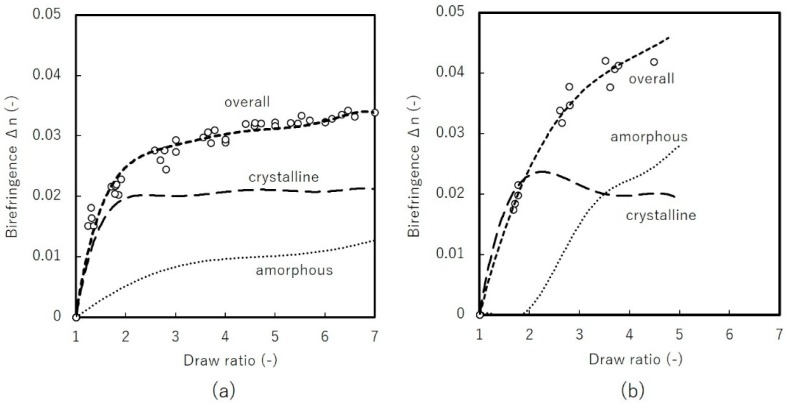
Birefringence vs. draw-ratio of uniaxial: (**a**) free-width (**b**) constrained-width stretched films at 100 °C and 120 °C with stretching speed of 1 m/min.

Figure 10 shows birefringence and the contributions from the crystalline and amorphous phase evaluated by WAXD. Degree of orientation and crystallinity were evaluated by Equation (2) to give the contribution from both crystalline and amorphous phase. Off-line birefringence measurements are plotted here, taking into account the shrinkage after stretching as the real DR; thus, they differ slightly from intended DR. In the case of uniaxial free-width stretching, we see that the contribution from the crystalline phase increase steeply up to DR = 2, then remains more or less constant. This is attributed to the offsetting contributions of increasing degree of orientation and decreasing crystallinity obtained by WAXD. The amorphous contribution plotted here, the remaining birefringence component, shows a growing contribution against DR, suggesting that the amorphous orientation increases monotonically.

Uniaxial constrained-width stretching shows different behavior. Birefringence appears not to approach the limit value under stretchable DR. Additionally, contributions from the crystalline phase gradually decrease, indicating that amorphous contributions become more significant. At the early stage of stretching until DR = 2, the amorphous contribution is negligible. This suggests that constraints along TD delay amorphous rearrangement. In both uniaxial stretching modes, the orientation of the amorphous phase is estimated to be significantly lower than that of the crystalline phase, considering that intrinsic birefringence of amorphous phase is estimated to be around 0.2 [26,27]. Note that this discussion assumes the absence of any significant relaxation in birefringence as observed by high-speed camera.

**Figure 11:**
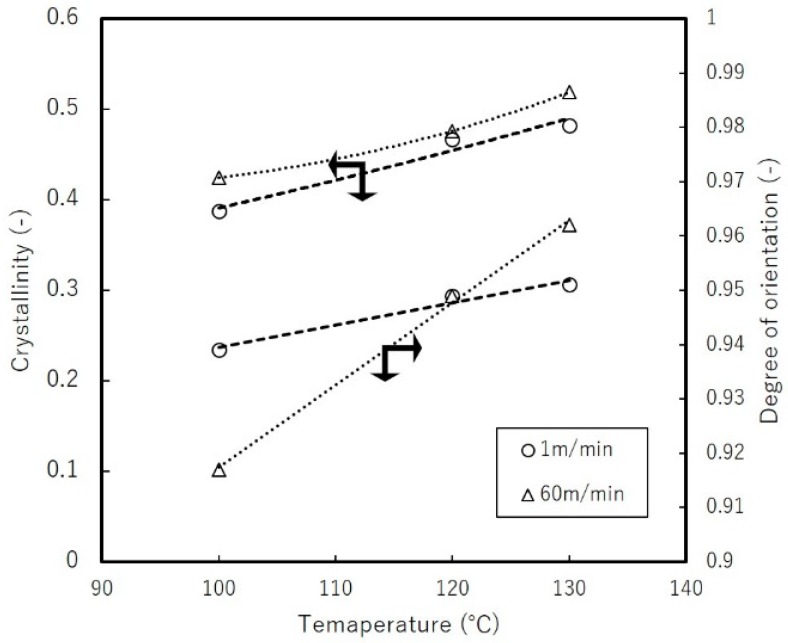
Crystallinity and orientation dependence on stretching temperature for uniaxial free-width stretched films by DR = 7.

Figure 11 compares the crystallinity of uniaxial free-width stretched film at different temperatures as evaluated by WAXD. It shows that higher temperatures and faster stretching speed tend to increase the crystallinity attained. The tendency of polymer chains to align is expected to increase at higher temperature, which would agree with our result.

**Figure 12:**
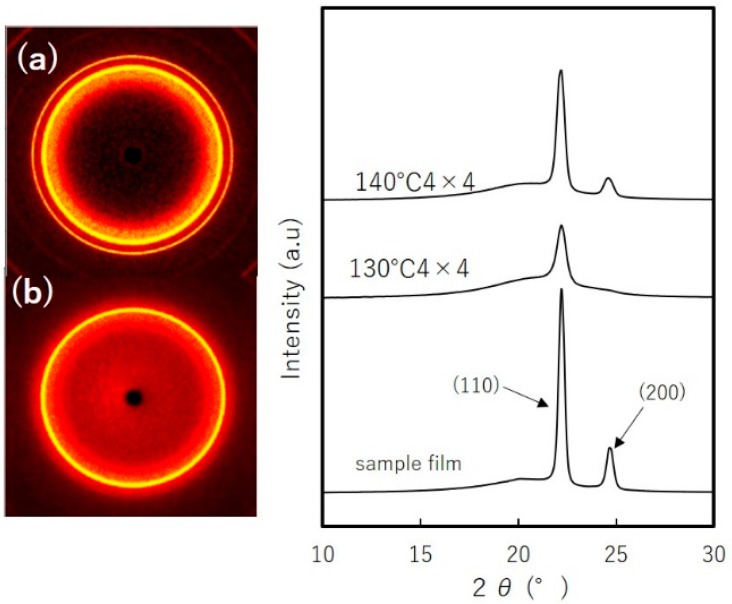
WAXD patterns of simultaneously biaxial stretched films 4 × 4 at: (**a**) 140 °C (**b**) 130 °C.

Figure 12 shows 2D WAXD patterns for simultaneous biaxial stretching. We see that the diffraction pattern is isotropic, with constant intensity along azimuthal angle. The crystallinity of the sample film as evaluated by WAXD is 44%. The crystallinity measured by DSC was 59%, but since crystallinity measurement of stretched film has difficulty due to thermal shrinkage, we refer to the measurement by WAXD. Stretching at 130 °C shows that the diffraction peak from (200) plane becomes negligible, while the contribution from the amorphous phase dominates. The crystallinity is evaluated to be 23%. The crystallinity of the film stretched at 140 °C is 43%, nearly equal to the sample film. To emphasize the differences between these films, we measured light transmittance. Film stretched at 130 °C exhibited transmittance of 47%, whereas for film stretched at 140 °C was 20%, a finding consistence with our results of crystallinity.

### 3.3. Thermal Analysis

#### 3.3.1. Dynamic Mechanical Analysis (DMA)

**Figure 13:**
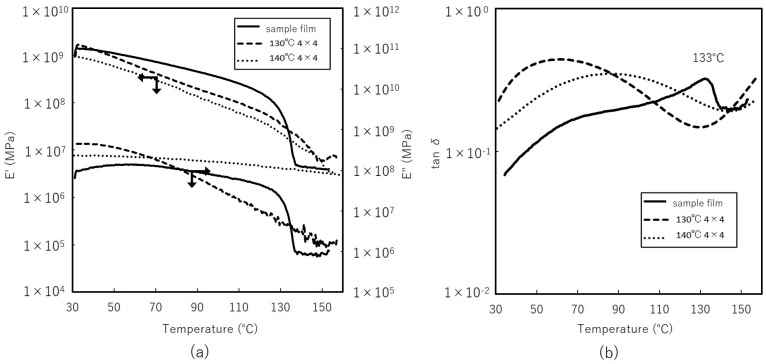
Dynamical mechanical analysis of simultaneous biaxially stretched films. (**a**) Storage and loss modulus (**b**) damping factor tan δ.

Figure 13 shows the results for storage modulus *E*’ and loss modulus *E*”, damping factor tan δ measurements. The storage moduli of the stretched films are more dependent on temperature than the film sample. The modulus of the sample film at *T*_m_, decreases significantly as the crystalline structure breaks down. Takayanagi model is known to evaluate modulus of semi-crystalline polymer which treats the material as composite of two-phase; crystalline and amorphous [28]. Difference in degree of crystallinity of the stretched films, may suggest different modulus behavior with temperature. However, stretched films exhibited similar linear changes on *E*’. Stable behaviors beyond *T*_m_ suggest that they maintain their form at high temperature. On loss modulus, film stretched at 130 °C with lower crystallinity decreased monotonically which reflects amorphous nature; at 140 °C, it remained stable with temperature. These contribute to the very broad curve for tan δ, with its peak shifting toward lower temperature. Inconsistency with simple physical model suggests taking into account the influence of orientation and morphology for more detailed analysis.

#### 3.3.2. Thermal Mechanical Analysis (TMA)

**Figure 14:**
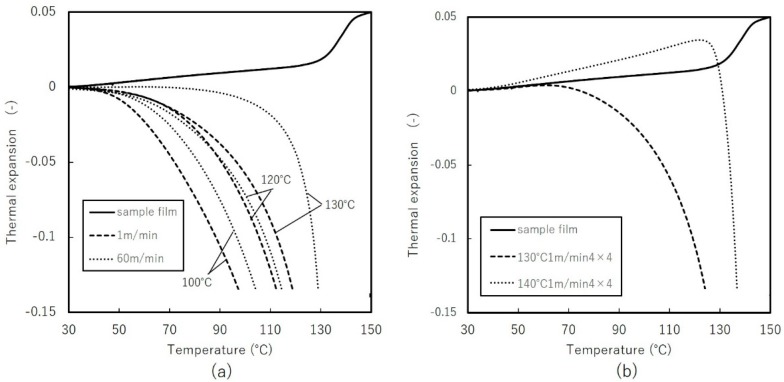
Thermal mechanical analysis of: (**a**) uniaxially free-width (**b**) simultaneous biaxially stretched films.

Figure 14 shows dimensional changes of uniaxially and biaxially stretched films with temperature. Uniaxially stretched films begin to shrink toward thermodynamically stable state. Comparing the results to Figure 11 shows that shrinkage behaviors correspond to degree of crystallinity and orientation. Fast stretching results in slightly higher crystallinity and greater stability with temperature changes. Biaxially stretched films initially exhibited positive thermal expansion at lower temperature, followed by dramatic shrinking. At low temperature, in-plane orientation and entanglement of polymer chains can interrupt shrinkage in one direction compared to uniaxial stretched films. Above certain temperatures, the mobility of the polymer chains, particularly in the amorphous phase, contributes to shrinkage, as observed with film stretched at 130 °C, at which crystallinity declines and early shrinkage appears. Requirements for lithium-ion battery separators include thermal shrinkage less than 5% after 60 min at 90 °C [1], biaxial stretching can be an effective stretching mode to satisfy such demands.

### 3.4. Tensile Tests

**Figure 15:**
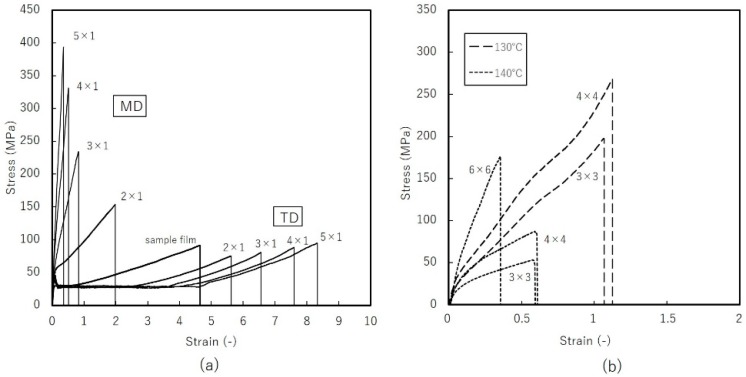
Tensile test results of: (**a**) uniaxial constrained-width stretched films at 120 °C (**b**) simultaneous biaxially stretched films at 140 °C with stretching speed of 1 m/min.

Figure 15 shows tensile test results for uniaxial constrained-width and simultaneous biaxially stretched films. The yield strength of the film sample was 30 Mpa; the strain-to-fracture ratio was about 4.5. Along the MD, strength increases in proportion to the DR of the film, while the strain-to-fracture ratio declines in inverse proportion to DR. Along the TD, the stress-strain curve exhibited neck formation until a strain of about 3–4. Stress at this stage is independent of the DR of the film. Strain to fracture increases with the DR of the film. For biaxially stretched films, breaking strength and strain increase significantly in films stretched at 130 °C. The greater ductility and strength can be attributed to the in-plane orientation of the amorphous phase (Figure 12). In sequential biaxial stretching, mechanical properties along the MD and TD are reversed between DR = 4 × 4 and 6 × 6. This coincided with the point at which stresses along the MD and TD overlap, suggesting that the stress-strain curve is an important predictor of isotropy. Biaxial stretching at 140 °C enabled to stretch up to high DR, but the strength of stretched films was not enhanced, as only DR larger than 4 × 4 could improve its strength.

## 4. Conclusions

We observed various film-stretching modes by retardation-measuring high-speed camera. Evaluations identified SOC in the 0.05–1.6 GPa^−1^ range. Uniaxial constrained-width stretching resulted in a broader range of SOC at low to high stretching speeds. Birefringence analysis showed at the early stage, constrained in the TD constrains orientation of the amorphous phase. WAXD and thermal analysis showed high crystallinity under high temperature at high stretching speeds. In biaxial stretching, a suitable stretching window occurred only near *T*_m_. Biaxial stretching below *T*_m_ showed significant changes in crystallinity and enhanced mechanical properties comparable to uniaxial stretching. Good thermal stability of biaxially stretched films were confirmed assuming energy storage application. Structural changes observed via retardation images captured with a retardation camera were consistent with results from optical micrographs. Our novel system tracking fast stretching process and in-plane retardation distribution is expected to extend laboratory scale research to industrial processes, emerging from trial and error processing to tailor-made processing.

## Figures and Tables

**Table 1:** Stretching mode, temperature and achieved draw-ratio in the experiment; stretching speeds are 1, 10, and 60 m/min for uniaxial mode, 1 m/min for biaxial mode.

Stretching Mode	Temperature (°C)			
	100	120	130	140
Uniaxial free-width	7 × 1	7 × 1	7 × 1	6 × 1
Uniaxial constrained-width	4 × 1	4 × 1	4 × 1	6 × 1
Simultaneous biaxial	-	Break	4 × 4	6 × 6
Sequential biaxial	-	Break	Break	6 × 6
